# Molecular and Functional Characterization of pheromone binding protein 1 from the Oriental Fruit Moth, *Grapholita molesta* (Busck)

**DOI:** 10.1038/s41598-018-20719-0

**Published:** 2018-02-02

**Authors:** Guohui Zhang, Jian Chen, Haili Yu, Xiaoli Tian, Junxiang Wu

**Affiliations:** 1 Institute of Entomology, College of Agriculture, Yangtze University, Jingzhou, Hubei 434025 China; 2 College of Life Science, Yangtze University, Jingzhou, Hubei 434025 China; 3 College of Plant Protection, North West A&F University, Yangling, Shaanxi 712100 China; 4 Wuwei Academy of Forestry Science, Wuwei, Gansu 733000 China

## Abstract

Pheromone binding protein (PBP) is thought primarily to bind and transport the sex pheromone in moths. The accumulated studies suggest that three PBPs were identified in moth species. In *Grapholita molesta*, the functions of GmolPBP2 and GmolPBP3 have been previously studied. However, the function of GmolPBP1 is still unclear. Furthermore, the *Cydia pomonella* sex pheromone Codlemone can act as a sex pheromone synergist of *G. molesta*. In *C. pomonella*, CpomPBP1 specifically bind the Codlemone. CpomPBP1 displays high identity with GmolPBP1 (70%), indicating that the two PBPs may share a similar 3D structure thus can bind the similar or same ligands. In this study, we explored the molecular and functional characterization of GmolPBP1. GmolPBP1, bearing the typical characteristics of Lepidopteran odorant binding proteins, was closest phylogenetically to CpomPBP1. Binding studies demonstrated that GmolPBP1 exhibited strong binding affinities with (*Z*)-8-dodecenyl alcohol, 1-dodecanol and Codlemone. Molecular docking showed that GmolPBP1 has different ligand recognition mechanism for the three ligands. Our results suggest that GmolPBP1 functions as recognizer of (*Z*)-8-dodecenyl alcohol and 1-dodecanol of the female sex pheromone blend, and may be the potential transporter of Codlemone, which contributes to the synergism of the pheromone response of *G. molesta* by Codlemone.

## Introduction

The pheromone communication system plays important roles in moth reproduction. They use this system to perceive sex pheromones for locating mating partners. Perception of sex pheromones is thought primarily to be mediated by small soluble proteins (15–17 kDa), the pheromone binding protein (PBPs), which are localized mainly in the lymph of sensilla trichodea on the moth antennae^1–3^. These proteins typically feature six conserved cysteine residues, which may form three disulfide bridges that are important to stabilizing the 3-D structure^4,5^. Insect PBPs are believed to bind and ferry hydrophobic sex pheromones across the aqueous sensillum lymph to the olfactory receptors (ORs) located on the olfactory receptor neurons (ORNs) where signal transduction is initiated^6–8^. Several PBPs have been shown to bind sex pheromone compounds^9–11^. Moreover, some studies have indicated that the absent of a PBP can alter chemosensory behavioral phenotypes or reduce the sensitivity of a sensory neuron to its odor ligand. In *Drosophila melanogaster*, the pheromone receptor OR67d depends on the PBP protein LUSH for responding to the pheromone *cis*-vaccenyl acetate (cVA). The *Drosophila* mutant PBP/LUSH has no sensitivity to its sex pheromone^12–14^. In addition, the *D. melanogaster* olfactory neurons expressing the *B.mori* pheromone receptor BmorOR1 can respond to the moth pheromone in the presence of any endogenous OBPs, however, when BmorOR1 and BmorPBP1 was co-expressed, the neurons showed greater sensitivity to the pheromone ligand^15^. In moth *Heliothis virescens*, when the *H. virescens* pheromone receptor HvirOR13 was expressed in the HEK293 cells, the cells showed very little specificity for all the pheromone components, but demonstrated significant sensitivity to the pheromone (*Z*)-11-Hexadecenal in the presence of HvirPBP2^16^. These results indicate that PBPs have important functions in the process of pheromone reception. They may bind and ferry distinct pheromones to the olfactory receptors and heighten the sensitivity of the olfactory receptor neurons to pheromones^17^.

Oriental fruit moth, *Grapholita molesta* (Busck), is an important pest of stone fruit and causes great economic losses in fruit production worldwide. Because of the disadvantages of pesticide control, an effective alternative control strategy is urgently required for management of this destructive fruit pest. Although sex pheromones are widely applied in oriental fruit moth control and monitoring, limited information is available about the molecular mechanisms of sex pheromone perception in *G. molesta*. The female sex pheromone blend of *G. molesta* comprises of four components^18^: (*Z*)-8-dodecenyl acetate, (*E*)-8-dodecenyl acetate, (*Z*)-8-dodecenyl alcohol and 1-dodecanol. The accumulated studies suggest that multiple PBPs were found in a single species, which strongly implies functional differentiation of these PBPs, possibly in discrimination among pheromone components^9,19–21^. In moth, whose PBPs were identified usually contain three PBP genes^22–26^. In our previous studies, three PBP genes (*GmolPBP1*, *GmolPBP2* and *GmolPBP3*) have been found from *G. molesta*^27^, and GmolPBP2 showed higher affinity to (*Z*)-8-dodecenyl acetate and (*E*)-8-dodecenyl acetate than (*Z*)-8-dodecenyl alcohol and 1-dodecanol, but GmolPBP3 showed poor affinity to all four sex pheromone components^28^. To further determine whether different PBPs selectively recognize distinct pheromone components, it is necessary to explore the binding affinities of the another PBP (GmolPBP1) to the sex pheromone components.

In addition, *G. molesta* and *Cydia pomonella* are two closely related and sympatric orchard pests in most parts of the world. The major sex pheromone component Codlemone (*E*, *E*-8, 10-Dodecadienol) for *C. pomonella* has been widely used in its management^29,30^. However, this compound when added to the *G. molesta* pheromone blend of (*Z*)-8-dodecenyl acetate (85.5%), (*E*)-8-dodecenyl acetate (5.5%) and (*Z*)-8-dodecenyl alcohol (9%), can act as a synergist by increasing trap catches of male *G. molesta* by two- to three-fold over the *G. molesta* blend alone^31^. Recent studies showed that Codlemone showed strong affinity to a *C. pomonella* PBP (CpomPBP1) but poor affinity to CpomPBP2, as well as indicated that Phe12 and Trp37 are two key residues in determining the binding affinity of Codlemone to CpomPBP1^32,33^. Our result indicated that Gmol PBP1 displays high identities on the amino acid sequence with CpomPBP1 (70%), phylogenetic analysis also revealed that GmolPBP1 is closest phylogenetically to CpomPBP1, indicating that these two PBPs may share a similar 3D structure thus binds with similar or same components of sex pheromone. The study on binding interaction between GmolPBP1 and Codlemone allows one to test whether or not there is correlative molecular support for the synergism of the pheromone response of *G. molesta* by Codlemone.

In this study, *G. molesta* PBP1 (Gmol PBP1) was cloned and expressed. We investigated the binding affinities of GmolPBP1 to the four sex pheromone components of *G. molesta* and a major sex pheromone component Codlemone of *C. pomonella*. In addition, we conducted a molecular docking to explore the binding modes between GmolPBP1 and ligands, as well as the potential key residues in the binding pockets of GmolPBP1. Our results not only help us better understand the functions of different PBPs in *G. molesta*, but also suggest that the protein GmolPBP1 may be the potential transporter of Codlemone in the olfaction system of *G. molesta*.

## Results

### Characteristics of the GmolPBP1 gene

**Figure 1:**
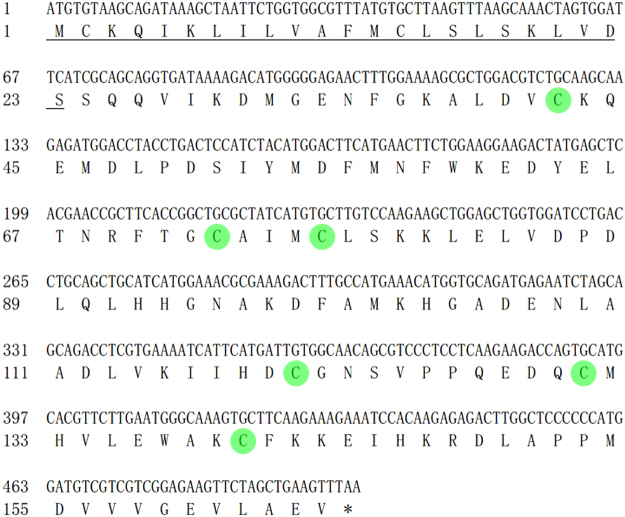
Nucleotide and predicted aa sequence of *GmolPBP1* from *Grapholita molesta*. The stop codon is marked by an asterisk. The signal peptide is underlined. The six conserved cysteines are circled with a green background.

According to the antennal transcriptome annotations and BLAST search, we designed a pair of specific primers and cloned the *GmolPBP1* by using reverse transcriptionpolymerase chain reaction (RT-PCR) from the antennal cDNA of *G. molesta*. The *GmolPBP1* gene contains a 498-bp open reading frame, which encodes a 165 amino acids protein. This predicted protein has a signal peptide of 23 amino acids, suggesting the solubility of GmolPBP1. The mature GmolPBP1 comprises 142 amino acids, with a molecular weight of 16,153 Da and an isoelectric point of 4.83 (Fig. 1). Moreover, GmolPBP1 possessed the common motif of insect OBPs: C1-X15-39-C2-X3-C3-X21-44-C4-X7-12-C5-X8-C6, in which the six conserved cysteine residues are thought to form three disulphide bridges (Fig. 1).

**Figure 2:**
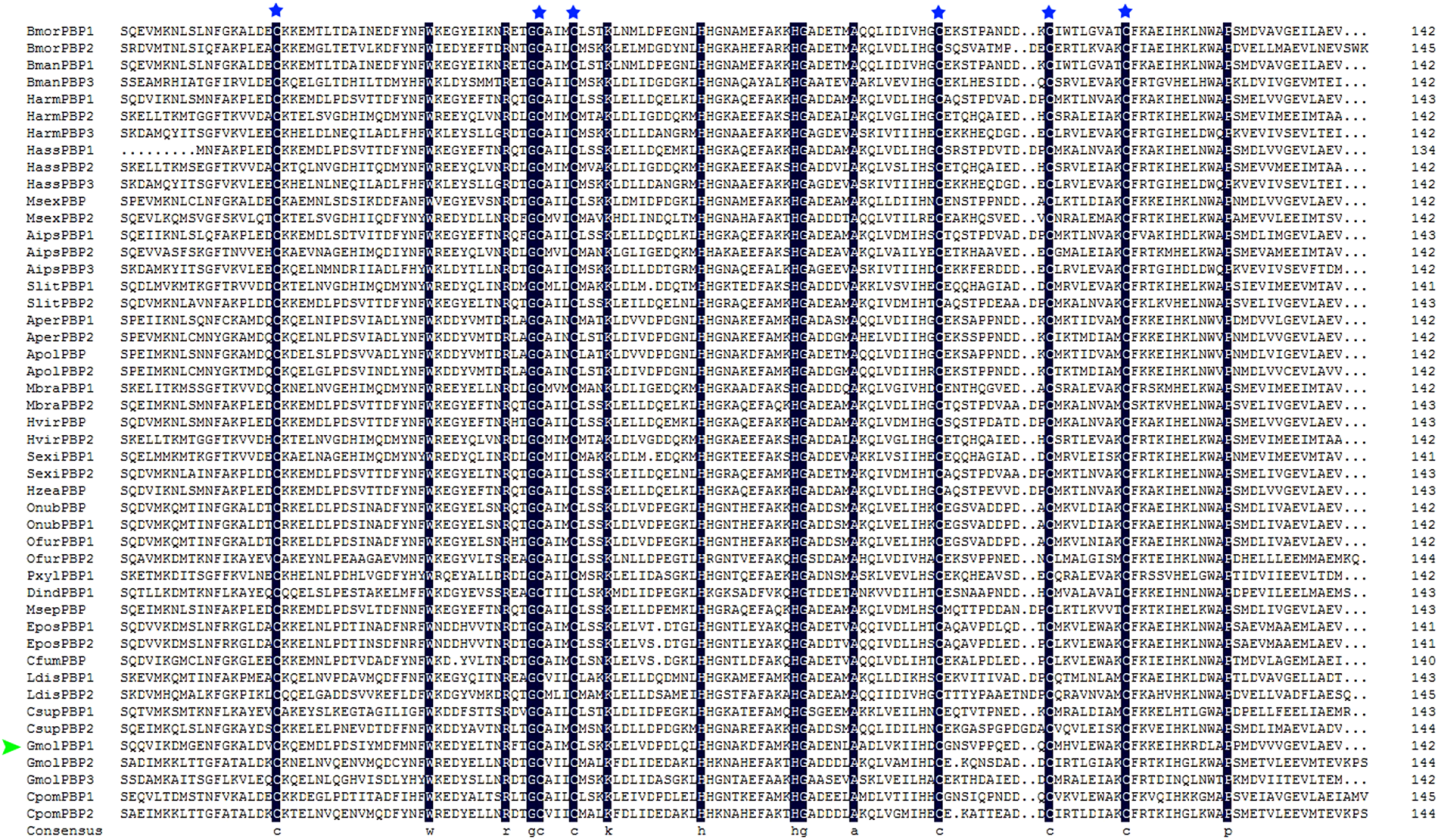
Alignments of the mature GmolPBP1 from *Grapholita molesta* with its orthologs from other Lepidopteran insects. The PBPs are: BmorPBP1 (*Bombyx mori*, X94987); BmorPBP2 (*Bombyx mori*, AM403100); BmanPBP1 (*Bombyx mandarina*, GQ246497); BmanPBP3 (*Bombyx mandarina*, GQ468570); HarmPBP1 (*Helicoverpa armigera*, HQ436362); HarmPBP2 (*Helicoverpa armigera*, HQ436360); HarmPBP3 (*Helicoverpa armigera*, AF527054); HassPBP1 (*Helicoverpa assulta*, AY864775); HassPBP2 (*Helicoverpa assulta*, EU316186); HassPBP3 (*Helicoverpa assulta*, DQ286414); MsexPBP (*Manduca sexta*, AF117593); MsexPBP2 (*Manduca sexta*, AF117588); AipsPBP1 (*Agrotis ipsilon*, JQ822240); AipsPBP2 (*Agrotis ipsilon*, JQ822241); AipsPBP3 (*Agrotis ipsilon*, JQ822242); SlitPBP1 (*Spodoptera litura*, DQ004497); SlitPBP2 (*Spodoptera litura*, DQ114219); AperPBP1 (*Antheraea Pernyi*, X96773); AperPBP2 (*Antheraea Pernyi*, AY301987); ApolPBP (*Antheraea polyphemus*, X17559); ApolPBP2 (*Antheraea polyphemus*, AJ277266); MbraPBP1 (*Mamestra brassicae*, AF051143); MbraPBP2 (*Mamestra brassicae*, AF051142); HvirPBP (*Heliothis virescens*, X96861); HvirPBP2 (*Heliothis virescens*, AM403491); SexiPBP1 (*Spodoptera exigua*, AY540316); SexiPBP2 (*Spodoptera exigua*, AY545636); HzeaPBP (*Helicoverpa zea*, AF090191); OnubPBP (*Ostrinia nubilalis*, GU828019); OnubPBP2 (*Ostrinia nubilalis*, GU826166); OfurPBP1 (*Ostrinia furnacalis*, GU828024); OfurPBP2 (*Ostrinia furnacalis*, GU828025); PxylPBP1 (*Plutella xylostella*, FJ201994); DindPBP1 (*Diaphania indica*, AB263115); MsepPBP (*Mythimna separate*, AB263112); EposPBP1 (*Epiphyas postvittana*, AF416588); EposPBP2 (*Epiphyas postvittana*, AF416587); CfumPBP (*Choristoneura fumiferana*, AF177644); LdisPBP1 (*Lymantria dispar*, AF007867); LdisPBP2 (*Lymantria dispar*, AF007868); CsupPBP1 (*Chilo suppressalis*, GU321120); CsupPBP2 (*Chilo suppressalis*, EU825762); GmolPBP2 (*Grapholita molesta*, KF365878); GmolPBP3 (*Grapholita molesta*, KF365879); CpomPBP1: (*Cydia pomonella*, see Tian and Zhang, 2016); CpomPBP2 (*Cydia pomonella*, JQ776635). Residues common to all PBPs are highlighted with dark blue background. Position of the six conserved cysteine residues are marked with blue stars. GmolPBP1 are marked with a green triangle.

**Figure 3:**
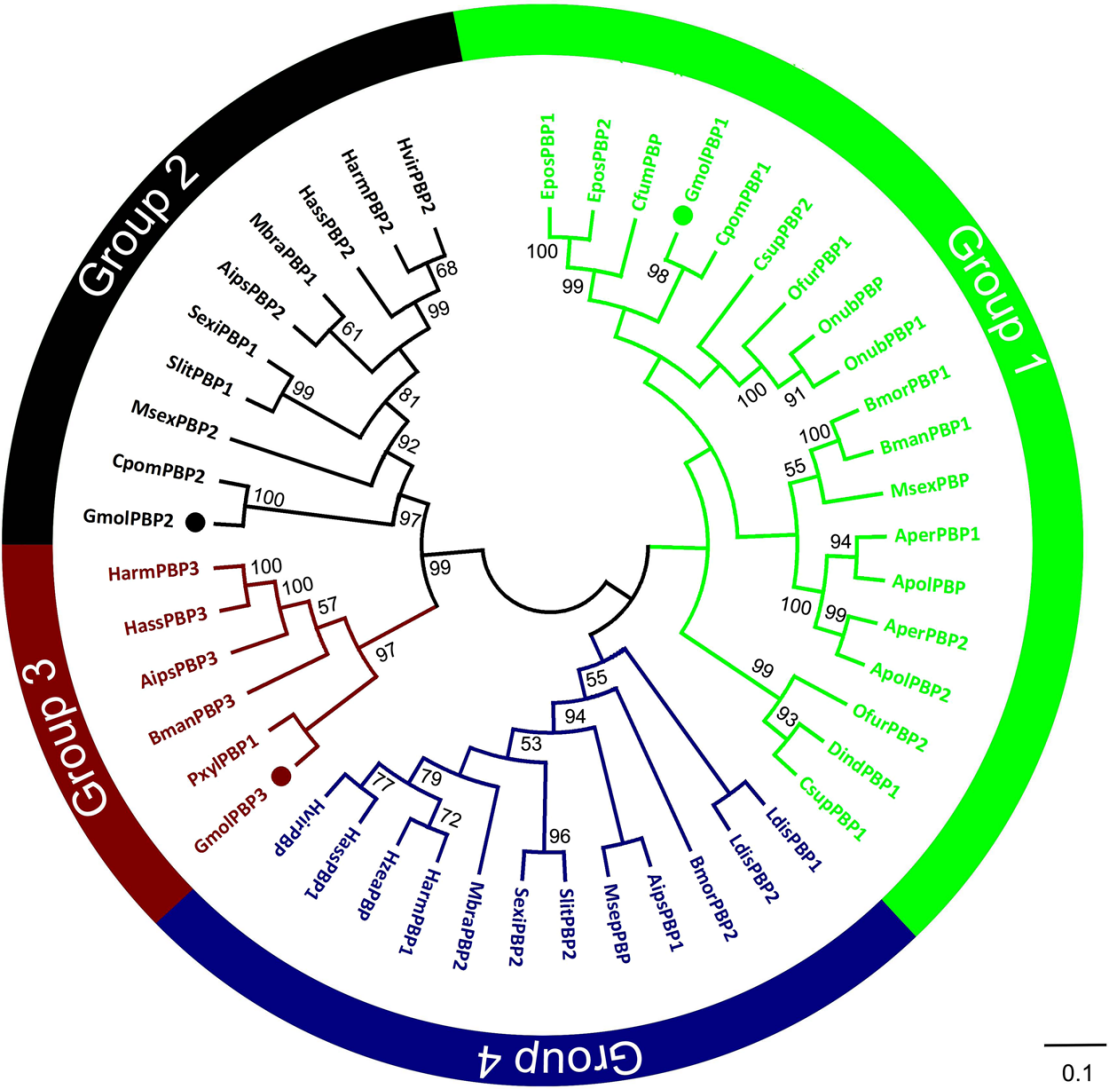
Neighbour-Joining tree of 47 PBP proteins of 24 Lepidoptera species. The GenBank accession numbers was list in Fig. 2. Bootstrap analysis used 1,000 replicates. The Points refer to the three *Grapholita molesta* pheromone binding proteins (PBPs).

The mature amino acid sequences of GmolPBP1 was aligned and compared with PBPs of other Lepidoptera (Fig. 2). GmolPBP1 exhibits a relatively low sequence similarity with other two GmolPBPs, with 44% for GmolPBP2 and 46% for GmolPBP3. However, GmolPBP1displays ~57 to 70% similarity with other Tortricid PBPs reported, including *E. postvittana*, *C. fumiferana* and *C pomonella*. The closest homolog of GmolPBP1 is CpomPBP1, with a sequence identity of 70%. We constructed a neighbor-joining tree consisting of 47 PBP protein sequences from 24 Lepidoptera species (Fig. 3). In this tree, these PBP sequences were distinguished into four subgroups. The three GmolPBPs belong to different subgroups: GmolPBP1 in Group 1, GmolPBP2 in Group 2 and GmolPBP3 in Group 3. No *G. molesta* homolog was found in Group 4 to date. Within the group 1, GmolPBP1 locates in the phylogenetic branch with the other Tortricid PBPs as stated above. GmolPBP1 is phylogenetically closest to Cpom PBP1 from *C. pomonella*, according with the fact that *G. molesta* and *C. pomonella* belong to the same tribe (Tortricidae: Olethreutinae: Grapholitini)^34^.

### Fluorescence binding assays

**Figure 4:**
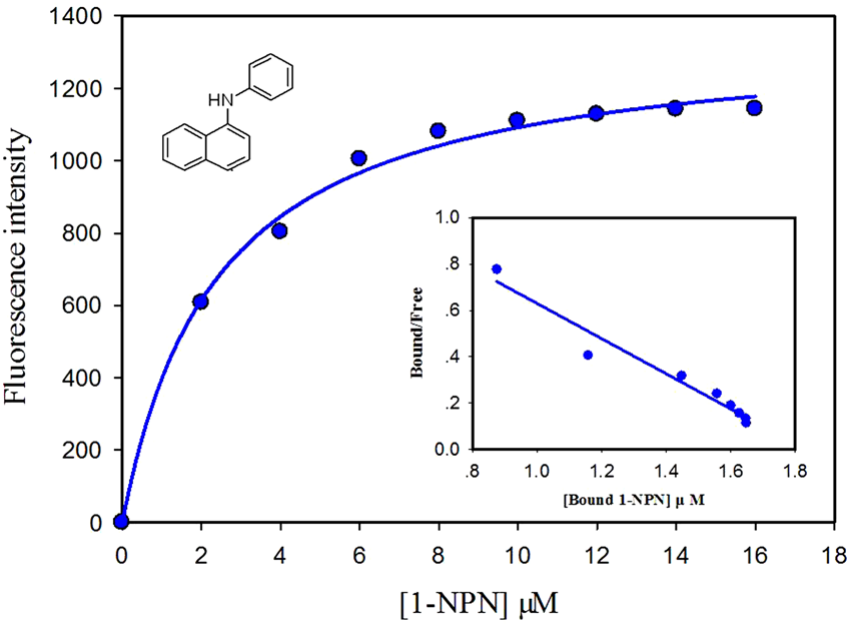
Binding curve of 1-NPN to GmolPBP1 and its resulting Scatchard plot (insert). The dissociation constant of CmolPBP1/1-NPN complex was 2.41 ± 0.25 μM.

To understand the binding properties of the GmolPBP1 with four sex pheromone components of *G. molesta* and the Codlemone of *C. pomonella*. We expressed and purified the protein (Figure S1). Then, a competitive binding assay was performed with 1-NPN as the fluorescence probe. The probe was excited at 337 nm and emission spectra were recorded between 370 and 500 nm. The fluorescent intensity of the 1-NPN alone was fairly low, then a strong blue shift, and peaked at about 410 nm with the presence of GmolPBP1. The intensity of this peak was used to determine the dissociation constant of the GmolPBP1/1-NPN complex. The fitted curve based on the intensity of this peak upon 1-NPN titration is shown in Fig. 4. The binding curve and Scatchard plot indicate a dissociation constant of 2.41 ± 0.25 μM, which was used to calculate the dissociation constants (K_i_) of different sex pheromone components in the competitive binding assy.

**Figure 5:**
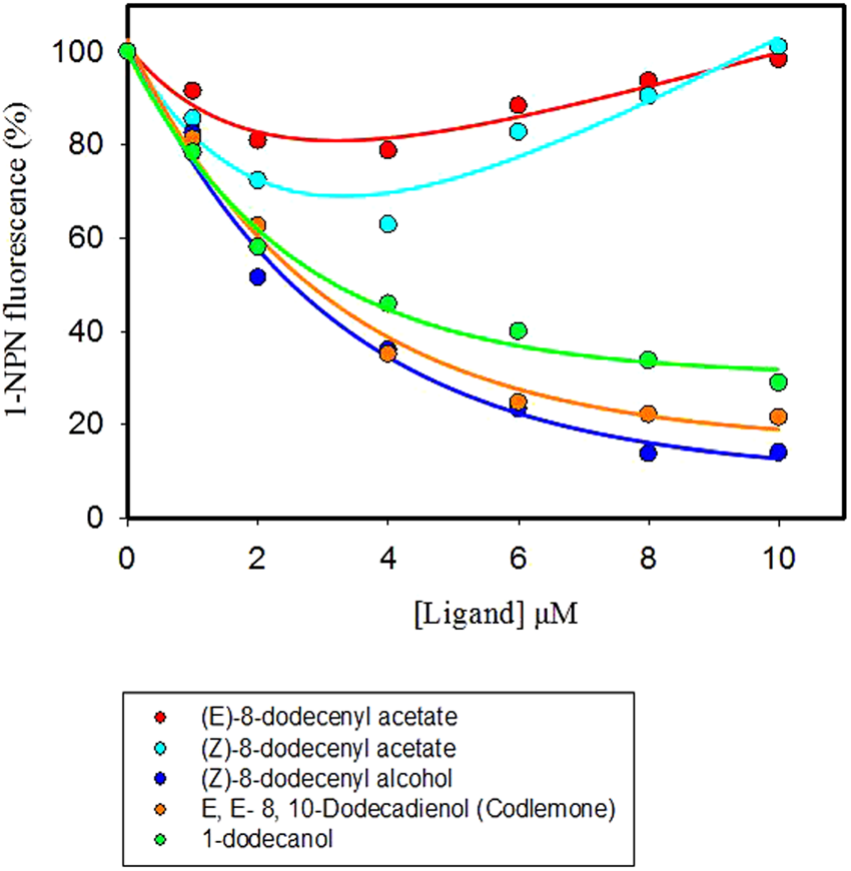
Fluorescence competitive binding curves of GmolPBP1 to (*Z*)-8-dodecenyl acetate, (*E*)-8-dodecenyl acetate, (*Z*)-8-dodecenyl alcohol, 1-dodecanol and Codlemone.

The displacement of 1-NPN by different sex pheromone components was measured for GmolPBP1 in the competitive binding assy. These ligands included the four sex pheromone components of *G. molesta* (*Z*)-8-dodecenyl acetate, (*E*)-8-dodecenyl acetate, (*Z*)-8-dodecenyl alcohol and 1-dodecanol as well as the *C. pomonella* Codlemone. The IC_50_ values and Ki values was shown in Table S2. The results of the competitive binding assay indicated that the three ligands, (*Z*)-8-dodecenyl alcohol, 1-dodecanol and Codlemone, present a strong affinity to GmolPBP1 (Fig. 5). The dissociation constants for these three compounds are 1.73 ± 0.31, 2.25 ± 0.63 and 1.88 ± 0.25 μM, respectively. However, the GmolPBP1 had fairly low binding affinities to (*Z*)-8-dodecenyl acetate and (*E*)-8-dodecenyl acetate, which could not displace half of the total 1-NPN from the GmolPBP1/1-NPN complex at a high concentration (Fig. 5).

### 3D model of GmolPBP1

**Figure 6:**
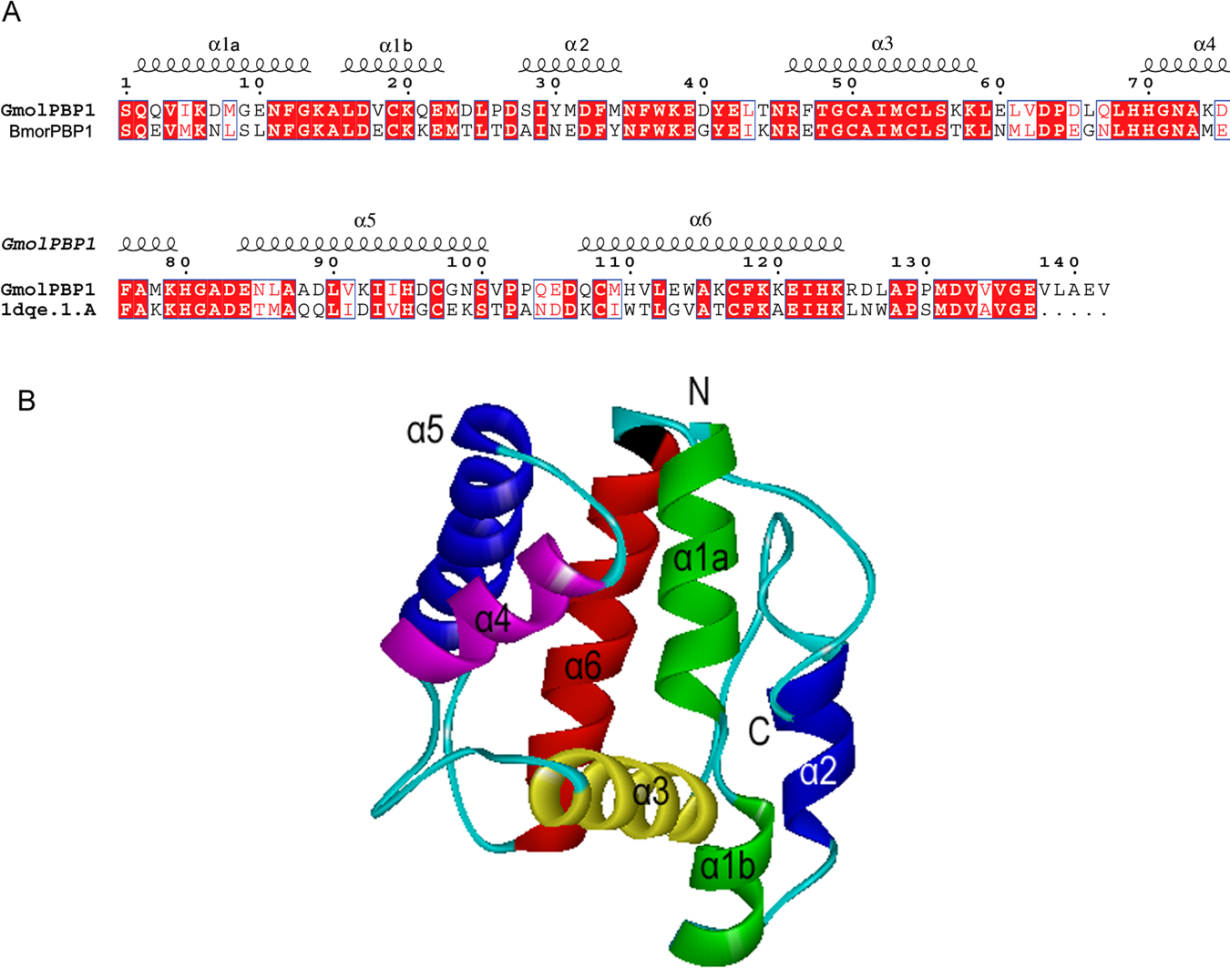
3D model of *Grapholita molesta* PBP1 (GmolPBP1). (**A**) Sequence alignment between GmolPBP1 and BmorPBP1. The seven α-helices are shown as squiggles. The identical residues are highlighted in white letters with a red background. Residues with similar Physico-chemical properties are shown in red letters. (**B**) 3D structure of GmolPBP1. N is the N-terminus, C is the C-terminus, and the seven helices are also labeled.

Using homology modeling, the target sequence must share sequence similarity more than 30% with the template^35–37^. In this study, we selected BmorPBP1 as the structural template and built the 3D structural model of GmolPBP1. As shown in Fig. 6A, GmolPBP1 shared 57% sequence identity with the selected template (BmorPBP1). 3D quality of the model was assessed by Pro-CHECK^38^ (http://services.mbi.ucla.edu/SAVES/Ramachandran/) (Fig. S2). The result revealed that 94.89% of all residues were in the favored regions, which were greater than the criterion for judging the rationality (90%). Moreover, the model of GmolPBP1 was verified by 3D-Profile^39^, the verified score of the GmolPBP1 model by 3D-Profile was 57.96, which was close to the expected high score of 61.95. All of these parameters suggest that the 3D structure of GmolPBP1 is reliable. As shown in Figs 3D and 6B structure of GmolPBP1 was composed of seven α-helices connected by loops. The seven α-helices located between residues 2–13 (α1a), 16–22 (α1b), 28–34 (α2), 46–58 (α3), 70–79 (α4), 84–100 (α4) and 107–124 (α6). Thereinto, α1a, α4, α5 and α6 formed the binding pocket whose wider end was capped by the α3, and the opposite narrow end of the pocket was open for the entry of ligands.

### Molecular docking

**Figure 7:**
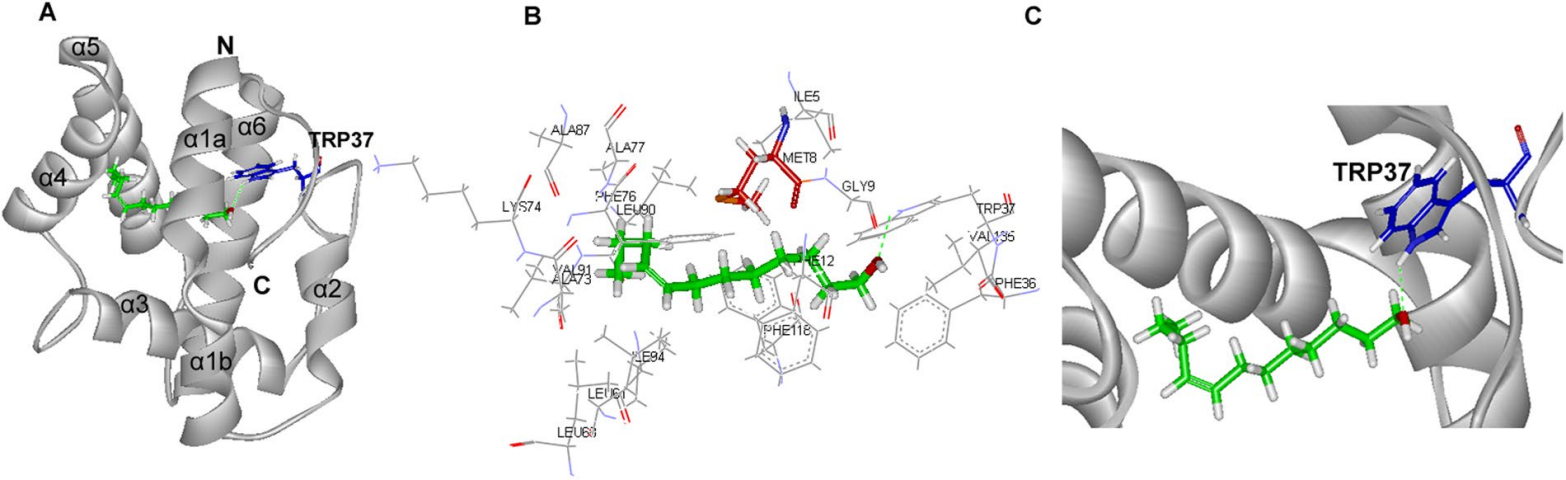
Molecular docking of GomlPBP1 to (*Z*)-8-dodecenyl alcohol. (**A**) Binding mode of GomlPBP1 with (*Z*)-8-dodecenyl alcohol. (*Z*)-8-dodecenyl alcohol presented as a green stick model with the hydroxyl oxygen in red. Blue stick represents Trp37 that forms hydrogen bond with (*Z*)-8-dodecenyl alcohol. (**B**) Diagram of the van der Waals interactions and hydrophobic interactions of (*Z*)-8-dodecenyl alcohol with key binding site residues. Residues shown as labeled drawing have a distance to (*Z*)-8-dodecenyl alcohol of less than 4 Å. Met8 is shown as red stick. (**C**) The orientation and conformation of (*Z*)-8-dodecenyl alcohol and Hydrogen bond reaction in the protein active area.

**Figure 8:**
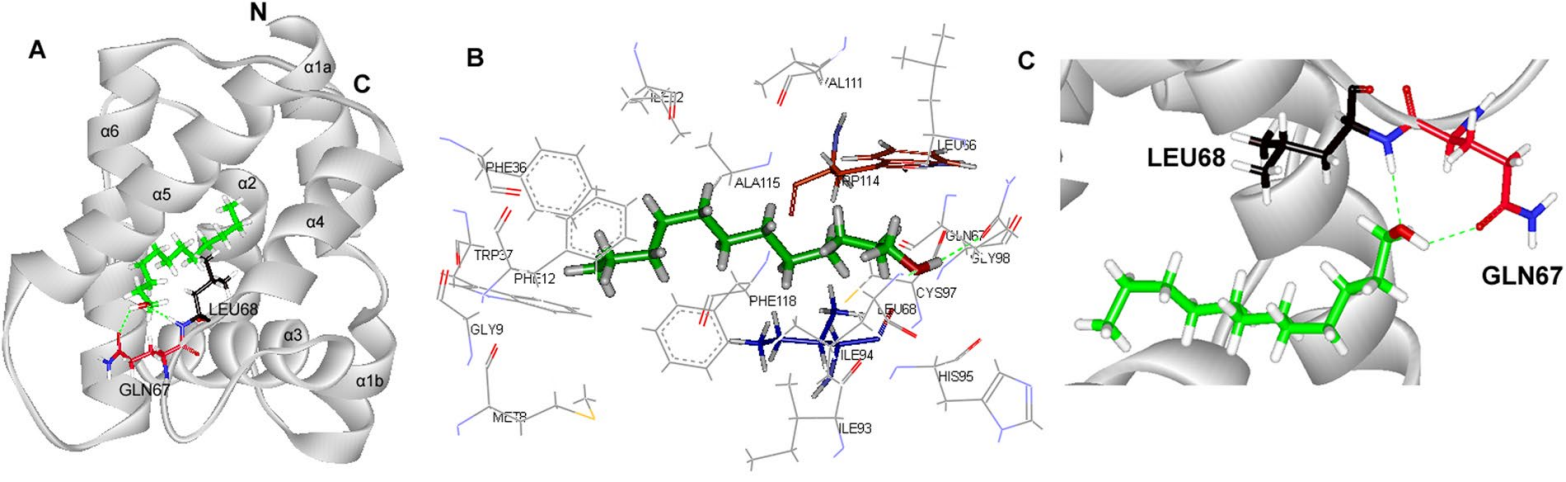
Molecular docking of GomlPBP1 to 1-dodecanol. (**A**) Binding mode of GomlPBP1 with 1-dodecanol. 1-dodecanol presented as a green stick model with the hydroxyl oxygen in red, which separately forms a hydrogen bonds with Gln67 (red stick) and Leu 68 (black stick). (**B**) Diagram of the van der Waals interactions and hydrophobic interactions of 1-dodecanol with key binding site residues. Residues shown as labeled drawing have a distance to 1-dodecanol of less than 4 Å. Ile 94 and Trp114 are shown as blue and red stick, respectively. (**C**) The orientation and conformation of 1-dodecanol and Hydrogen bond reaction in the protein active area.

**Figure 9:**
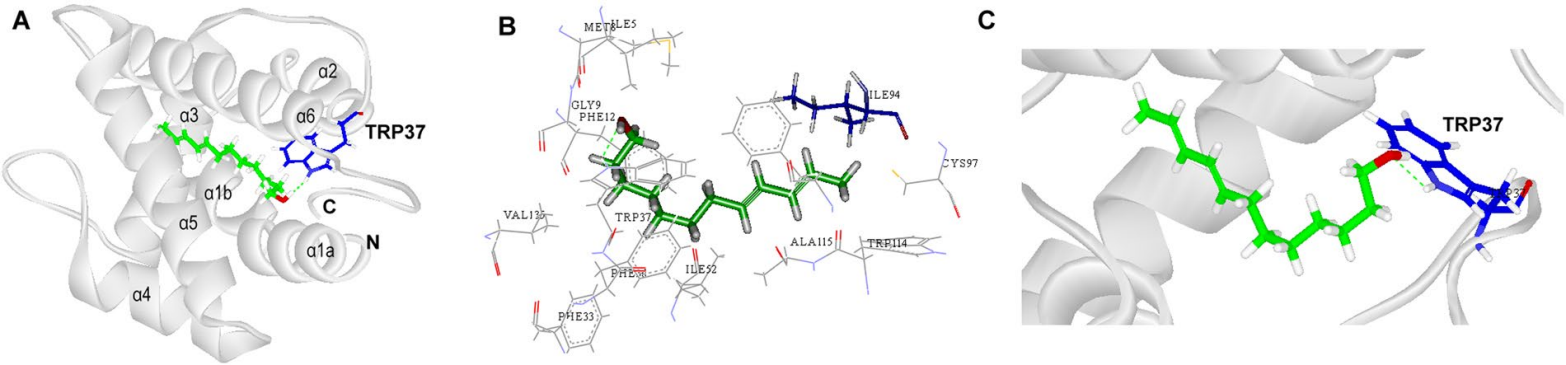
Molecular docking of GomlPBP1 to Codlemone. (**A**) Binding mode of GomlPBP1 with Codlemone. Codlemone presented as a green stick model with the hydroxyl oxygen in red. Blue stick represents Trp37 that forms hydrogen bond with Codlemone. (**B**) Diagram of the van der Waals interactions and hydrophobic interactions of Codlemone with key binding site residues. Residues shown as labeled drawing have a distance to Codlemone of less than 4 Å. Ile 94 is shown as dark blue stick. (**C**) The orientation and conformation of Codlemone and Hydrogen bond reaction in the protein active area.

To explore the binding mode of GmolPBP1 with the sex pheromone components which have strong binding affinity with GmolPBP1. Three compounds ((*Z*)-8-dodecenyl alcohol, 1-dodecanol and Codlemone) were picked to dock with the 3D structure of GmolPBP1. CDOCKER molecular docking produced ten likely conformations for each GmolPBP1/ligand complex. The best conformation of complex was selected by CDOCKER energy and the binding mode. The result was shown in Figs 7, 8 and 9. Based on the molecular docking study, hydrogen bonds were the main linkage between GmolPBP1 and the three tested compounds. (*Z*)-8-dodecenyl alcohol and Codlemone possessed same hydrogen bond interaction with GmolPBP1: The hydroxyl oxygen of the two compounds formed hydrogen bonds with the side chain of Trp 37 (Figs 7C and 9C). Different from the two compounds above, 1-dodecanol formed two hydrogen bonds within the binding pocket of GmolPBP1 as shown in Fig. 8C. In the GmolPBP1/1-dodecanol complex, the oxygen atom and hydrogen atom from the hydroxyl group of 1-dodecanol formed hydrogen bonds separately with HN atom from the main chain of Leu68 and oxygen atom from the side chain of Gln67 (Fig. 8C). These hydrogen bonds acted like a holder which strongly fixed the ligands in the binding site.

Besides the hydrogen bonds, van der waals and hydrophobic interactions were also the important linkages between GmolPBP1 and the test ligands. The residues within 4.0 Å to (*Z*)-8-dodecenyl alcohol, 1-dodecanol and Codlemone were represented in Figs 7B, 8B and 9B, respectively. In GmolPBP1/(*Z*)-8-dodecenyl alcohol complex, these residues within 4.0 Å to the ligand: Ile5, Met8, Gly9, Phe12, Phe36, Trp37, Leu61, Leu68, Ala73, Lys74, Phe76, Ala77, Ala87, Leu90, Val91, Ile94, Phe118 and Val135. Thereinto, the distance between Met8 and (*Z*)-8-dodecenyl alcohol was 1.39 Å, which was the shortest distance over which van der waals interactions were formed (Fig. 7B). In GmolPBP1/1-dodecanol complex, they are Met8, Gly9, Phe12, Phe36, Trp37, Ile52, Leu66, Gln67, Leu68, Ile93, Ile94, His95, Cys97, Gly98, Val111, Trp114, Ala115 and Phe118. Within this complex, 1-dodecanol formed van der waals interactions with Ile 94 and Trp114 as well, with the distance 1.21 Å and 1.16 Å, respectively (Fig. 8B). In GmolPBP1/Codlemone complex, they are Ile5, Met8, Gly9, Phe12, Phe33, Phe36, Trp37, Ile52, Ile94, Cys97, Trp114, Ala115, Phe118 and Val135. Among these redidues, Ile94 formed van der waals interactions with Codlemone whose distance was 1.02 Å (Fig. 9B).

## Discussion

Insect PBPs are believed to function as sex pheromone transporters, thus playing an important role in pheromone recognition. The accumulated studies suggest that multiple PBPs were found in a single species, which strongly implies functional differentiation of these PBPs, possibly in discrimination among pheromone components. In moth, whose PBPs were identified usually contain three PBP genes^22–26^. In *G. molesta*, the molecular characterization and binding properties of two PBPs (GmolPBP2 and GmolPBP3) have been previously studied^28^. In present study, we cloned and characterized the gene that encodes GmolPBP1 from the antennae of *G. molesta*. The mature proteins contain 142 amino acids, and possess all the hallmarks of the OBP family: an acidic isoelectric point, a major hydrophobic domain, a signal peptide and six conserved cysteine residues^40^. The identification of GmolPBP1 leads to a total of three *GmolPBPs* in this species to date. This allows us to further study the function of GmolPBP1 and compare the functionality differences among the *G. molesta* PBPs.

Sequence similarity analysis has shown that all these Lepidoptera PBPs have a 6-conserved cysteine motif and share high overall sequence similarity (62.6%) (Fig. 2). This supporting the hypothesis that the moth PBPs share a common ancestor and then later diverged by gene duplication events after specialization^41,42^. Phylogenetic analysis revealed their sequence diversities: these PBP sequences were divided into four subgroups. The three GmolPBPs are classified into different subgroups: GmolPBP1 in Group 1, GmolPBP2 in Group 2 and GmolPBP3 in Group 3 (Fig. 3). This indicated that each GmolPBP may form different 3D structures thus binding with different sex pheromone components of *G. molesta*. Moreover, GmolPBP1 is closely clustered with CpomPBP1 in Group 1 and GmolPBP2 is closely clustered with CpomPBP2 in Group 2 (Fig. 3), indicating the two PBP genes (*GmolPBP1* and *GmolPBP2*) may emerge from a gene duplication event and the duplication occurred before the separation of the two different Olethreutinae species. Among four groups (1, 2, 3 and 4) we only found a homolog in Group 1, 2 and 3. It is possible the *G. molesta* only have three different PBPs, just like many species in Noctuidae. However, we cannot exclude that the expression of *G. molesta* member of Group 4 are too low to be cloned in the antennae.

The ligand-binding property of OBP is very important for the functional study of OBP^43–47^. PBPs have been shown to bind sex pheromone in many species^9–11^. In *G. molesta*, the sex pheromone of this species is a four-component blend^18^, (*Z*)-8-dodecenyl acetate, (*E*)-8-dodecenyl acetate, (*Z*)-8-dodecenyl alcohol and 1-dodecanol. In this regard, three GmolPBPs may be tuned to detect a specific component(s) of the sex pheromone blend. In previous study, the binding properties of the two *G. molesta* PBPs (GmolPBP2 and GmolPBP3) had been studied. The result showed that GmolPBP2 exhibited strong affinity to (*Z*)-8-dodecenyl acetate and (*E*)-8-dodecenyl acetate, with the K_i_ values of 1.09 ± 0.04 and 1.10 ± 0.05 μM, respectively. GmolPBP3 displayed poor affinity to all four sex pheromone components^28^. In present study, the fluorescence binding assays showed that GmolPBP1 displayed high binding affinities only for the two sex pheromone components of *G. molesta* (*Z*)-8-dodecenyl alcohol and 1-dodecanol, with K_i_ values of 1.73 ± 0.31 and 2.25 ± 0.63 μM, respectively. Based on the information above, we considered that the *G. molesta* PBPs may be able to discriminate or selectively recognize pheromone components, and the GmolPBP1 and CmolPBP2 may play main role in pheromone discrimination.

*G. molesta* and *C. pollonella* are two closely related and sympatric orchard pests. Codlemone is major sex pheromone component of *C. pollonella*^29,30^. Interestingly, this compound when added to the *G. molesta* pheromone blend, can act as a synergist by increasing trap catches of male *G. molesta*^31^. Recent studies showed that the Codlemone showed strong affinity to a *C. pollonella* PBP (CpomPBP1) but poor affinity to CpomPBP2, indicating CpomPBP1 play an important role in the perception of Codlemone^32,33^. Our result indicated that GmolPBP1 is closest phlylogenetically to CpomPBP1 and the two PBP proteins share 70% sequence similarity. Based on the information above, we speculate that the two PBPs may share a similar 3D structure thus binds with similar or same components of sex pheromone. To test the hypothesis, the fluorescence binding assay was carried out to determine the affinity between GmolPBP1 and Codlemone. The result indicated that GmolPBP1 displayed much higher binding affinities for Codlemone with Ki values of 1.88 ± 0.25 μM. This result suggests that the protein Gmol PBP1 may be the potential transporter of Codlemone in the olfaction system of *C. pollonella* and contributes to the synergism of the pheromone response of *G. molesta* by Codlemone, at least in the process of peripheral olfactory signal transduction.

Based on the results of our fluorescence binding assay, (*Z*)-8-dodecenyl alcohol, 1-dodecanol and Codlemone was picked as the ligands to dock with the 3D structure of GmolPBP1. Those ligands all characterized by a same chain length (12 carbons) and with hydroxyl group at one end of each molecule. Previous studies have demonstrated that this functional group tends to form hydrogen bond with the PBP residus^48,49^. The hydrogen bonds have been confirmed as the primary linkage between proteins and ligands in several insect OBPs^46,50–52^. However, there are quite different in hydrogen bond formation between the ligands with saturated aliphatic chain ((*Z*)-8-dodecenyl alcohol and Codlemone) and the ligand with unsaturated chain (1-dodecanol). The docking experiments suggested that (*Z*)-8-dodecenyl alcohol and Codlemone possessed same hydrogen bond interaction with GmolPBP1. The hydrogen bond was established between the oxygen atom derived from the hydroxyl group of the compound and the hydrogen atom from the side chain of Trp37 at the entrance of the binding cavity (Figs 7A,B and 9A,B). The hydrogen bonds formed at the entrance of the OBP binding cavity are crucial for ligand-binding specificity, as previously observed in LUSH^50^, ApolPBP1^53^, CpomPBP1^33^ and BmorPBP^48^. For CpomPBP1, the Codlemone also formed hydrogen bond with Trp37 at the entrance of the binding cavity, indicating that GmolPBP1 and CpomPBP1 are most likely share the similar ligand recognition mechanism for Codlemone. Comparing our results with the other reports, we speculated that the Trp37 of GmolPBP1 is one of the crucial binding sites involved in the recognition of (*Z*)-8-dodecenyl alcohol and Codlemone. Different from the two compounds above, 1-dodecanol formed hydrogen bonds separately with Leu68 and Gln67, and the two amino acid residues located at the bottom of the binding pocket (Fig. 8A,B). This inferred that GmolPBP1 may have the different ligand recognition mechanism between the two sex pheromone compounds ((*Z*)-8-dodecenyl alcohol and 1-dodecanol), even though the two compounds share a very similar structure. Although the computational simulations need to be confirmed by crystal structure of proteins, the results of the molecular docking presented here provide a foundation for further site-directed mutagenesis study on the molecular mechanisms of pheromome-GmolPBP1 interactions.

## Methods

### RNA extraction and first- strand cDNA synthesis

The *G. molesta* used in the present study were reared at 25 ± 1 °C (15: 9, L:D) on an artificial diet in the laboratory of the College of Plant Protection, North West A&F University, Yangling, China. Antennae were collected from three- to four day-old moths and immediately frozen in liquid nitrogen. Total RNA isolated using RNAiso Plus reagent (TaKaRa, Shiga, Japan), following the manufacturer’s instructions. Single strand cNDA synthesis was performed with Oligo (dT)_18_ primer using M-MLV reverse transcriptase (TaKaRa) according to the recommended protocols.

### Molecular cloning and sequencing

According to the unigenes of the OBPs annotated from the antennal transcriptome database of *G. molesta*, a pair of gene-specific primers were designed to amplify the coding region of the corresponding GmolPBP1 cDNA (GmolPBP1-forward: 5′-ATGTGTAAGCAGATAAAGC-3′ and GmolPBP1-reverse: 5′-TTAAACTTCAGCTAGAACTTCTCC-3′). The PCR conditions: were 94 °C for 3 min, followed by 30 cycles of 94 °C for 30 sec, 50 °C for 30 sec, 72 °C for 1 min, and final extension for 72 °C for 10 min. The amplification product purified with the DNA purification kit (Tiangen Biotechnologies, Beijing, China) cloned into the pGEM-t easy vector (promega Madison, WI, USA) and then transformed into DH5α competent cells (TransGen Biotechologies, Beijing, China). The positive clones were checked by PCR amplification and restriction enzyme digestion and were sequenced at Genscript Biotech Company (Genscript Nanjing China).

### Sequence analysis and phylogenetic tree construction

The SignalP 4.1 program ((http://www.cbs.dtu.dk/services/SignalP/)) was used to determine signal peptides. The ExPASy server ((http://web.expasy.org/compute_pi/)) was used to calculate the molecular weight and isoelectric point of mature protein. The alignment of PBP sequences was made using Clustal X 1.83. The Neighbor joining method was used to build the phylogenetic tree by using MEGA 4.0 software. Bootstrap analysis used 1,000 replications.

### Expression vector construction

The gene-specific primer 5′> CCGGAATTCTCGCAGCAGGTGATAAAAG <3′ (forward) 5′> CCCAAGCTTTTAAACTTCAGCTAGAAC <3′ (reverse) (*Eco*R I and *Hin*d III restriction sites are underlined) were designed to clone cDNA that encode mature GmolPBP1 protein from the antennal cDNA. The PCR product was first cloned into pGEM-T easy vector (Promega). Positive clones were confirmed by sequencing. The plasmids of Positive clones ware digested by *Eco*R I and *Hin*d III QuickCut ® restriction enzymes (TaKaRa). The digested product was separated by agarose gel electrophoresis. The expected band was purified on the agarose gel and ligated into the empty expression vector pET-30a (+) (Novagen, Darmstadt, Germany), which had been digested with same restriction enzymes. The resulting recombinant plasmid was sequenced to confirm that it encoded the mature protein.

### Recombinant GmolPBP1 expression and purification

The recombinant vectors were transformed into BL21 (ED3) competent cells. The positive clones were confirmed by sequencing. Protein expression was induced at 28 °C for 5 h with 0.5 mM isopropyl-β-D-thiogalactopryranoside (IPTG) when the culture OD_600_ reached 0.6. The bacterial cells were harvested by centrifugation (8000 g, 10 min). The cellular pellets were resuspended in the lysis buffer (50 mM Tris-HCI, pH 8.0, 50 mM NaCI, 0.5% TritonX-100, 2 mg/mLIysozyme) and then sonicated (10 s, 5 passes). After centrifugation, the recombinant proteins were present as inclusion bodies. The inclusion bodies were solubilized and refolded following by the previously reported protocols^54,55^. Purification of the protein was accomplished by a Ni-NTA His·bind Resin column (7 sea Pharmatech Co., Shanghai, China), according to the manufacturer’s protocol. The His-tag was removed by digestion with recombinant enterokinase (Novoprotein, Shanghai, China), and the target proteins were purified again by the column mentioned above and analyzed by sodium dodecyl sulphate polyacrylamide gel electrophoresis (SDS-PAGE). The concentration of the purified proteins was measured using BCA protein assay kit (Beyotime, Shanghai, China).

### Fluorescence binding assay

Emission fluorescence spectra were performed on an F-4600 fluorescence Spectrophotometer (Hitachi, Japan) in a 1 cm light path quartz cuvette with 10 nm slit width for both excitation and emission. The excitation wavelength was set at 337 nm and emission spectra were recorded between 370 and 500 nm. The fluorescent probe N-phenyl-1-naphthylamine (1-NPN) and the ligands used in the binding assay were dissolved in spectrophotometric-grade methanol to yield a 1-mM stock solution (for the sources of these chemicals, see Table S1). To determine the dissociation constant between 1-NPN and GmolPBP1, 2 μM GmolPBP1 dissolved in 20 mM Tris-HCl pH 7.4 was titrated with 1 mM 1-NPN to final concentrations of 2–16 μM. The fluorescence intensities at the maximum fluorescence emission between 370 and 500 nm were plotted against the concentrations of added 1-NPN. The curve was linearized using Scatchard plots. The concentrations of bound 1-NPN were evaluated from the values of fluorescence intensity assuming that the protein was 100% active with a stoichiometry of 1:1 (protein: ligand) at saturation. The affinities of the selected ligands were measured by competitive binding assays by adding the ligands from 1 to 10 μM into the 1-NPN/GmolPBP1 solution (both at 2 μM). The emission spectra were recorded between 370 and 500 nm and the maximum fluorescence intensities were plotted against the ligand concentrations after normalization relative to the intensity at zero ligand. For these competitor ligands, the dissociation constants were computed from the corresponding IC_50_ values using the quation: K_i_ = [IC_50_]/(1 + [1-NPN]/K_1-NPN_), where [1-NPN] is the free concentration of 1-NPN and K_1-NPN_ is the dissociation constant of the protein complex/1-NPN. All values reported were collected in three independent measurements.

### 3D modeling and molecular docking

After searching for the current PDB (http://www.rcsb.org) with the amino acid sequence of GmolPBP1 being a probe, the crystalline structure of a *B. mori* PBP BmorPBP1 (PDB ID: 1DQE, Chain A, resolution 1.8 Å) was selected as a template. The Sequences alignment between target and the template were conducted by using Clustal X 1.83. The resulting alignment was submitted to the SWISS-MODEL server (https://swissmodel.expasy.org/interactive) for comparative structural modeling^35^. The optimum alignment, as determined by the lowest QMEAN4 score and Anolea score, was selected. The P_RO_-CHECK (http://services.mbi.ucla.edu/SAVES/Ramachandran/)^38^ and the P_ROFILES_-3D^39^ were further used to estimate the modeling rationality. Using the built model, a docking program, CDOCKER^56^, was applied to analyze the potential binding mode between GmolPBP1 and the ligands. The 3D structures of chemicals were sketched and refined by using CHARMm force-field in Discovery Studio 2.0. The top ten docking poses ranked by CDOCDER energy were retained for subsequent finding of the most optimal binding mode.

### Data Availability statement

All data generated or analysed during this study are included in this published article (and its Supplementary Information files).

## Electronic supplementary material

Supplementary material
