# Supplementary material for: Molecular and Functional Characterization of pheromone binding protein 1 from the Oriental Fruit Moth, Grapholita molesta (Busck)
Source: Sci Rep. 2018 Feb 2;8:2276. doi: 10.1038/s41598-018-20719-0 (PMC5797111; doi:10.1038/s41598-018-20719-0)
Supplement: Supplementary file 1 — Supplementary material [file 41598_2018_20719_MOESM1_ESM.doc]

**Molecular and Functional Characterization of pheromone binding protein 1 from the Oriental Fruit Moth, *Grapholita molesta* (Busck)**

**Guohui Zhang^1*^, Jian Chen^1^, Haili Yu^4^, Xiaoli Tian^2^ & Junxiang Wu^3^**

^1^ Institute of Entomology, College of Agriculture, Yangtze University, Jingzhou, Hubei 434025, P. R. China

^2^ College of Life Science, Yangtze University, Jingzhou, Hubei 434025, P. R. China

^3^College of Plant Protection, North West A&F University, Yangling, Shaanxi 712100, P. R.China.

^4^ Wuwei Academy of Forestry Science, Wuwei, Gansu 733000, P. R. China

**Corresponding author:**

Guohui Zhang

College of Agriculture, Yangtze University, Jingzhou, Hubei 434025, P. R. China

Telephone: +86-0176-8066314

Fax: +86-0176-8066314

Email: [ghzhang84@sina.com](mailto:ghzhang84@sina.com)

**Supplementary information**

| Table S1. Chemicals used in competitive binding assay | | | | | |
| --- | --- | --- | --- | --- | --- |
| Name | Structure | Molecular weight | Formula | Purity | Source |
| N-Phenyl-1-naphthylamine | 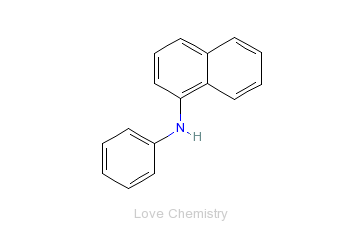 | 219.28 | C_16_H_13_N | >98.0%(GC) | TCI |
| (Z)-8-dodecenyl acetate | 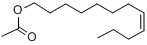 | 226.36 | C14H26O2 | >95.0%(AR) | BedoukianResearch |
| (E)-8-dodecenyl acetate | 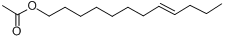 | 226.36 | C14H26O2 | >95.0%(AR) | BedoukianResearch |
| (Z)-8-dodecenyl alcohol | 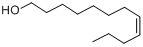 | 200.34 | C12H24O | >98.0%(AR) | BedoukianResearch |
| 1-dodecanol | 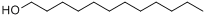 | 186.34 | C12H26O | >99.0%(GC) | BedoukianResearch |
| Codlemone | 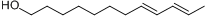 | 182.30 | C12H22O | >96.0%(GC) | Dr. Ehrenstorfer |

| Table S2. Binding affinities of ligands to recombinant *Grapholita molesta* PBP1 (GmolPBP1) | | |
| --- | --- | --- |
| Ligands | IC_50_ (μM) | K_i_ (μM) |
| Pheromone components of *Grapholita molesta* |  |  |
| (*Z*)-8-dodecenyl acetate | — | — |
| (*E*)-8-dodecenyl acetate | — | — |
| (*Z*)-8-dodecenyl alcohol | 2.46±0.43 | 1.73±0.31 |
| 1-dodecanol | 3.20±0.89 | 2.25±0.63 |
| Pheromone components of *Cydia pomonella* |  |  |
| Codlemone | 2.66±0.35 | 1.88±0.25 |
| IC_50_: ligand concentration displacing half of the initial fluorescence intensity of the GmolPBP1/1-NPN complex. The The dissociation constant (K_i_) were calculated from the IC_50_ value. “—” means that IC_50_ and K_i_ values were not calculated. | | |

**Figure S1. Expression and purification of *Grapholita molesta* PBP1 (GmolPBP1).** M: Protein molecular weight markers; 1, 2: a expression vector that was not inserted the target gene was used as control before and after induction by IPTG, respectively; 3, 4: the crude bacterial extracts before (lane 3) and after (lane 4) induction by IPTG, respectively; 5: the purified protein of GmolPBP1 with His-tag; 6: Repurification of GmolPBP1 after His-tag removal by recombinant enterokinase.

**Figure S2. The analysis of GmolPBP1 by Pro-CHECK.**

**Fig. S1**

**
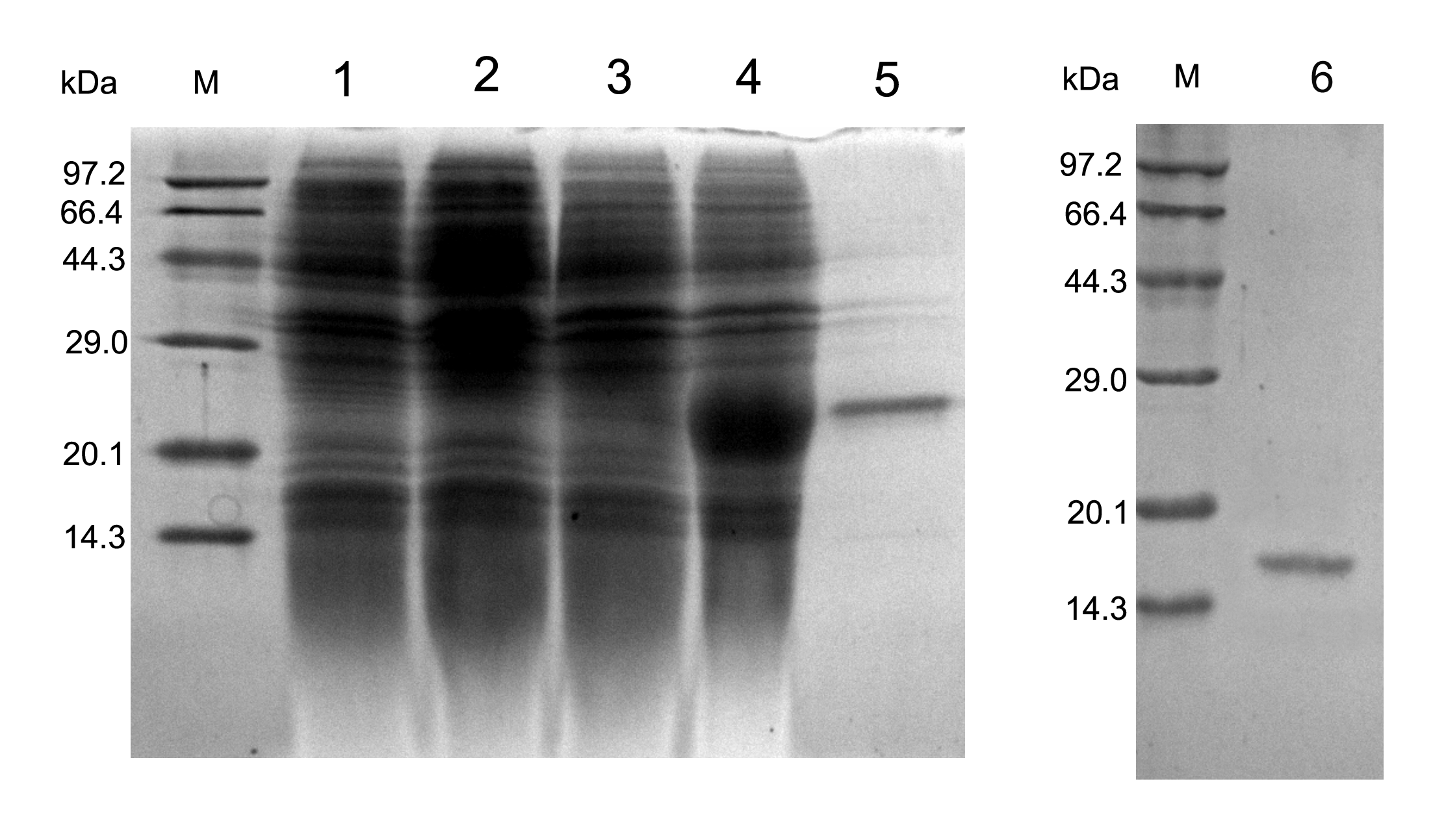
**

**Fig. S2**

**
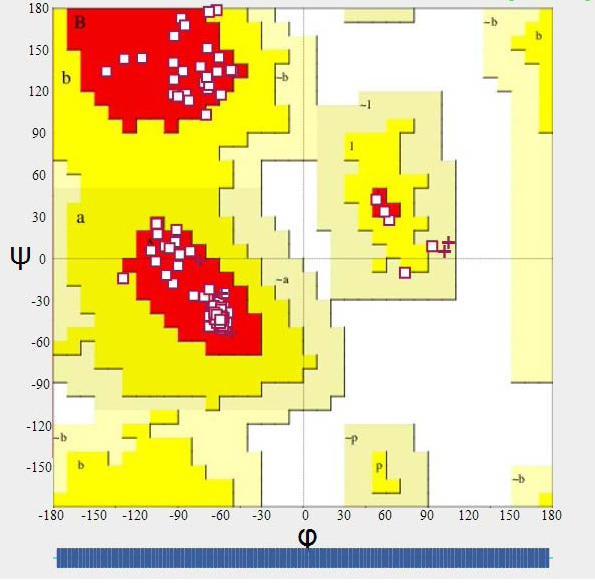
**
